# A Point in Prognosis

**Published:** 1884-04

**Authors:** B. E. Hadra


### A POINT IN PROGNOSIS.

There is a phenomenon presenting itself in some diseases of children which seems to me to be of more importance in connection with prognosis than generally known.

In exhaustive diseases, such as diarrhœa, typhoid fever, and others, after having for days persistently refused nourishment, the child suddenly swallows with avidity whatever is offered, food or medicine indiscriminately. Even Quinine will be taken as readily as sugar. Such an occurrence is generally hailed with delight by the interested bystanders, but in reality it is a very untoward symptom. In my experience it frequently warrants an unfavorable prognosis.

An explanation of this sudden change may perhaps be found in the cessation of cerebral function through the want of nutrition or of stimulation.

Combined with this behavior is often found the Cheyne-Stokes breathing, and this coincidence goes far to support the above explanation, as this respiratory disorder has been traced also to the want of stimulation of the respiratory centres.—*B. E. Hadra, M. D., Am. Jour. of Obstetrics, etc.*

